# Constitutively active transforming growth factor β receptor 1 in the mouse ovary promotes tumorigenesis

**DOI:** 10.18632/oncotarget.10149

**Published:** 2016-06-17

**Authors:** Yang Gao, David F. Vincent, Anna Jane Davis, Owen J. Sansom, Laurent Bartholin, Qinglei Li

**Affiliations:** ^1^ Department of Veterinary Integrative Biosciences, College of Veterinary Medicine and Biomedical Sciences, Texas A&M University, College Station, TX, USA; ^2^ Centre de Recherche en Cancérologie de Lyon, INSERM U1052, CNRS UMR5286, Lyon, France; ^3^ Cancer Research UK Beatson Institute, Garscube Estate, Glasgow, United Kingdom

**Keywords:** TGFβ, TGFBR1, tumor, ovary, mouse model

## Abstract

Despite the well-established tumor suppressive role of TGFβ proteins, depletion of key TGFβ signaling components in the mouse ovary does not induce a growth advantage. To define the role of TGFβ signaling in ovarian tumorigenesis, we created a mouse model expressing a constitutively active TGFβ receptor 1 (TGFBR1) in ovarian somatic cells using conditional gain-of-function approach. Remarkably, these mice developed ovarian sex cord-stromal tumors with complete penetrance, leading to reproductive failure and mortality. The tumors expressed multiple granulosa cell markers and caused elevated serum inhibin and estradiol levels, reminiscent of granulosa cell tumors. Consistent with the tumorigenic effect, overactivation of TGFBR1 altered tumor microenvironment by promoting angiogenesis and enhanced ovarian cell proliferation, accompanied by impaired cell differentiation and dysregulated expression of critical genes in ovarian function. By further exploiting complementary genetic models, we substantiated our finding that constitutively active TGFBR1 is a potent oncogenic switch in mouse granulosa cells. In summary, overactivation of TGFBR1 drives gonadal tumor development. The TGFBR1 constitutively active mouse model phenocopies a number of morphological, hormonal, and molecular features of human granulosa cell tumors and are potentially valuable for preclinical testing of targeted therapies to treat granulosa cell tumors, a class of poorly defined ovarian malignancies.

## INTRODUCTION

Sex cord-stromal tumors are derived from the granulosa, theca, and/or stromal fibroblast components of the ovary and represent ~8% of all categories of ovarian tumors [1]. Granulosa cell tumors which arise from ovarian granulosa cells [2] are most common sex cord-stromal tumors and account for ~ 5% of ovarian malignancies [1]. The molecular etiology of sex cord-stromal tumors is poorly defined, partially because of its rarity and the fact that research efforts in ovarian cancer research field have been predominantly focused on tumors of epithelial cell origin, the major type of ovarian tumors [1]. Although the prognosis of granulosa cell tumors is often better than that of epithelial tumors, a serious complication is the risk of prolonged recurrence (i.e., relapse), a significant cause of death in patients [3]. Treatment strategy for granulosa cell tumors is generally based on that for epithelial tumors which have distinct disease etiology, arguing for the need of developing tailored treatment options for this type of tumors.

Genetically modified mouse models are powerful tools in cancer research. To date, there are several mouse models for sex cord-stromal tumors [4–12], such as mice with targeted deletion of α inhibin (*Inha*) [4], *Smad1/5* [5], bone morphogenetic protein (BMP) type 1 receptors [6], and forkhead box O1/3 (*Foxo1/3*) and phosphatase and tensin homolog (*Pten*) [12] and constitutive activation of wingless-type MMTV integration site (WNT)/β-catenin [7, 8]. The findings of *Inha* as a tumor suppressor gene specific for the gonad and adrenal and the inhibitory function of BMP receptors and SMADs in ovarian tumor formation reveal the importance of the transforming growth factor β (TGFβ) superfamily in gonadal carcinogenesis [4–6].

TGFβ superfamily members play critical roles in the development of reproductive system and cancer [13, 14]. TGFβ ligands (i.e., TGFβs 1-3) signal through a heteromeric complex consisting of type 2 (TGFBR2) and type 1 (TGFBR1) receptors and intracellular SMAD proteins, which comprise receptor regulated SMADs (SMAD2/3 and SMAD1/5/8) and a common SMAD (i.e., SMAD4). Activation of SMAD2/3 and SMAD1/5/8 is associated with the transduction of TGFβ and BMP signaling, respectively [15]. TGFβ signaling generally acts as tumor suppressor *via* inhibiting cell proliferation during the early stage of tumor development. However, deletion of a number of key TGFβ signaling components (e.g., TGFβ1, TGFBR1, SMAD2/3, and SMAD4) alone in the ovary does not induce tumor formation [16–19], challenging TGFβ signaling as essential tumor suppressor in the ovary. In contrast to the involvement of BMP signaling (BMP type 1 receptors and BMP-responsive SMAD1/5/8) in ovarian tumor development [5, 6], the role of TGFβ signaling in the ovary remains elusive.

This study is therefore to identify the role of TGFβ signaling activation in the pathogenesis of ovarian tumors using conditional gain-of-function approach. We performed morphological, hormonal, and molecular analyses to determine the relevance of TGFBR1 constitutively active mice as a model for ovarian granulosa cell tumors.

## RESULTS

### Generation of mice harboring a constitutively active TGFBR1 in the ovary

**Figure 1:**
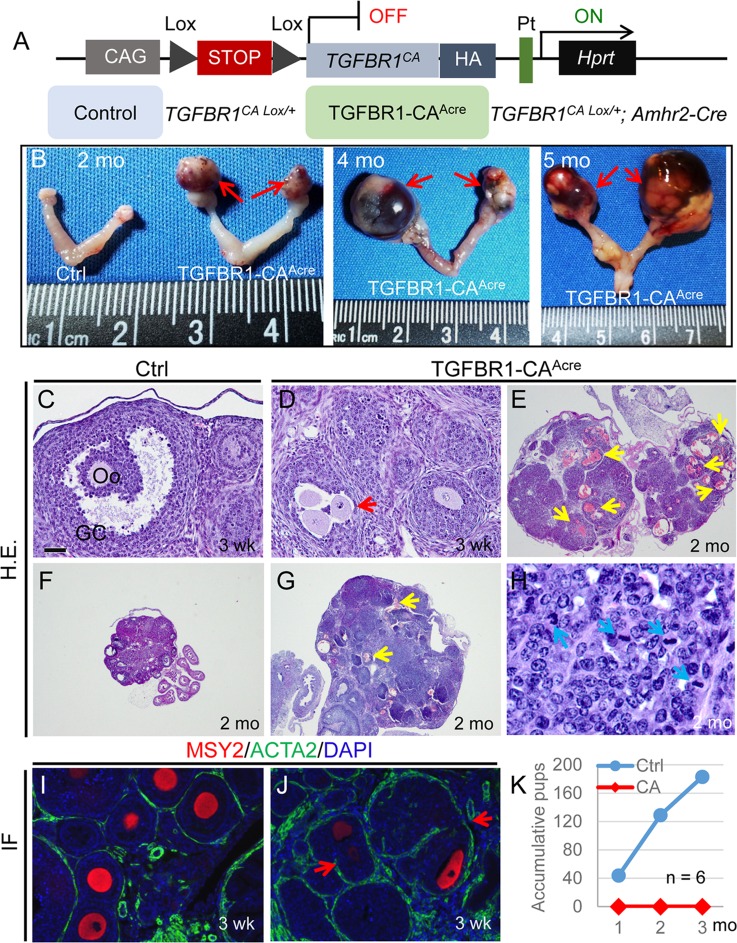
Constitutive activation of TGFBR1 in the mouse ovary using *Amhr2-*Cre leads to ovarian tumor development **A.** Schematic illustration of the latent constitutively active *TGFBR1* allele. Upon *Amhr2*-Cre mediated recombination, the stop sequence was removed, and the *TGFBR1*^CA^ was expressed in the ovary. Mice harboring the constitutively active *TGFBR1* were termed TGFBR1-CA^Acre^ (*TGFBR1*CA ^Lox/+^; *Amhr2*-Cre), and *TGFBR1*CA ^Lox/+^ mice were used as controls. *Pt*, *Hprt* promoter; *HA*, hemagglutinin tag; *CAG*, composite constitutive CAG (human cytomegalovirus enhancer and chicken beta-actin). **B.** Representative macroscopic images of ovarian tumors in TGFBR1-CA^Acre^ mice at 2 (*n* = 16), 4 (*n* = 7), and 5 (*n* = 6) months of age. Red arrows indicate ovarian tumors. **C.**-**J.** Histological and immunofluorescence analyses of ovaries from control (C, F, and I) and TGFBR1-CA^Acre^ mice (D, E, G, H, and J). Panel (H) represents a higher power image for panel (G). Note the presence of follicle-like structures containing multiple oocytes (D and J; red arrows) and the altered follicular structure in the TGFBR1-CA^Acre^ ovaries *versus* controls (C, F, and I), as was demonstrated by H&E staining and double immunofluorescence of ACTA2 (green) and MSY2 (red). Oo, oocyte; GC, granulosa cells. Yellow arrows indicate multifocal hemorrhage within follicle-like structures, while blue arrows indicate mitotic figures. DAPI was used to counterstain the nucleus. Scale bar is representatively depicted in (C) and equals 10 μm (H), 40 μm (C, D, I, and J), and 400 μm (E-G). **K.** Fertility defects in TGFBR1-CA^Acre^ mice. The TGFBR1-CA^Acre^ mice were sterile during a 3 month fertility test. Data represent accumulative pup numbers per month. *n* = 6.

A constitutively active TGFBR1 (*TGFBR1^CA^*), where three missense mutations (i.e., T204D and L193A/P194A) constitutively activate the TGFBR1 kinase [20] and prevent the binding of the TGFBR1 inhibitor FK506 binding protein 1A (FKBP12) [21], was constructed as described [22]. The *TGFBR1*^CA^ transgene was knocked into the hypoxanthine-guanine phosphoribosyltransferase (*Hprt*) locus, with a STOP sequence flanked by two Lox sites [22]. This allele has been successfully expressed in several compartments, including pancreas, liver, T lymphocytes, embryos, and uterus [23–26]. Upon Cre-mediated recombination, the STOP codon was removed, leading to the expression of constitutively active TGFBR1 under the control of the ubiquitous CAG (human cytomegalovirus enhancer and chicken beta-actin) promoter (Figure 1A). The *TGFBR1*^CA Lox/Lox^ mice were crossed with mice harboring anti-Mullerian hormone receptor type 2 (*Amhr2*)-Cre (Figure S1A and B; TGFBR1-CA^Acre^). Expression of *Amhr2*-Cre in mouse ovarian granulosa cells was verified by X-gal staining of ovaries from *Gt(ROSA)26Sor*; *Amhr2*-Cre reporter mice [27] (Figure S1D).

### Validation of mice with enhanced TGFβ signaling in the ovary

As evidence of recombination of *TGFBR1*^CA^ conditional allele in the *Amhr2*-Cre expressing tissues, PCR amplified a recombined band with the expected size in the ovaries of TGFBR1-CA^Acre^ mice, but not those of controls (Figure S2A). Consistently, expression of *TGFBR1*^CA^ mRNA transcripts was detected in the TGFBR1-CA^Acre^ ovary *versus* controls by both quantitative and conventional PCR analyses (Figure S2B and C). Furthermore, the presence of TGFBR1^CA^ fusion protein was confirmed in TGFBR1-CA^Acre^ ovaries by western blot using an anti-hemagglutinin (HA) antibody (Figure S2D). To further validate this model, we demonstrated increased levels of phosphorylated SMAD2, an indicator of TGFβ signaling activity, in ovarian tissues of TGFBR1-CA^Acre^ mice (Figure S2E). Coinciding with TGFβ signaling activation, expression of TGFβ target genes including TGFβ-induced (*Tgfbi*), serine/cysteine peptidase inhibitor clade E member 1 (*Serpine1*), connective tissue growth factor (*Ctgf*), and *Smad7* was increased in the ovaries of TGFBR1-CA^Acre^ mice (Figure S2F). Therefore, we successfully created a mouse model that harbors a constitutively active TGFBR1 in the ovary.

### Constitutive activation of TGFBR1 in the ovary promotes tumorigenesis

To determine the phenotypic consequence of constitutive activation of TGFBR1, we examined ovaries of control and TGFBR1-CA^Acre^ mice at various developmental stages by macroscopic, histological, and immunohistochemical analyses using antibodies against alpha smooth muscle actin (ACTA2; green) and Y box protein 2 (MSY2; red) [28] to mark normal theca layers and oocytes, respectively. Strikingly, gross ovarian tumors were prominent in TGFBR1-CA^Acre^ mice examined at 2 months of age (Figure 1B). The tumors progressed rapidly and the phenotype was exacerbated with age (Figure 1B), leading to the death of TGFBR1-CA^Acre^ mice. Tumor formation in the TGFBR1-CA^Acre^ ovary was accompanied by a disruption of follicular development, with loss of follicle boundary and presence of multiple oocytes containing follicles (Figure 1D and 1J; red arrows), compared with age-matched controls (Figure 1C and 1I). The neoplastic cells were arranged in lobules with multiple layers of cuboidal cells forming follicle-like structures (Figure 1G) that contained mitotic figures (Figure 1H; blue arrows). Neoplastic cells were cuboidal, with round to oval nuclei (Figure 1H). Multifocally, there was hemorrhage within these follicle-like structures (Figure 1E and 1G; yellow arrows). These results suggest that the neoplasms belong to sex cord-stromal tumors, reminiscent of ovarian granulosa cell tumors. As expected, the TGFBR1-CA^Acre^ females were sterile (Figure 1K).

### Molecular characterization of ovarian tumors

**Figure 2:**
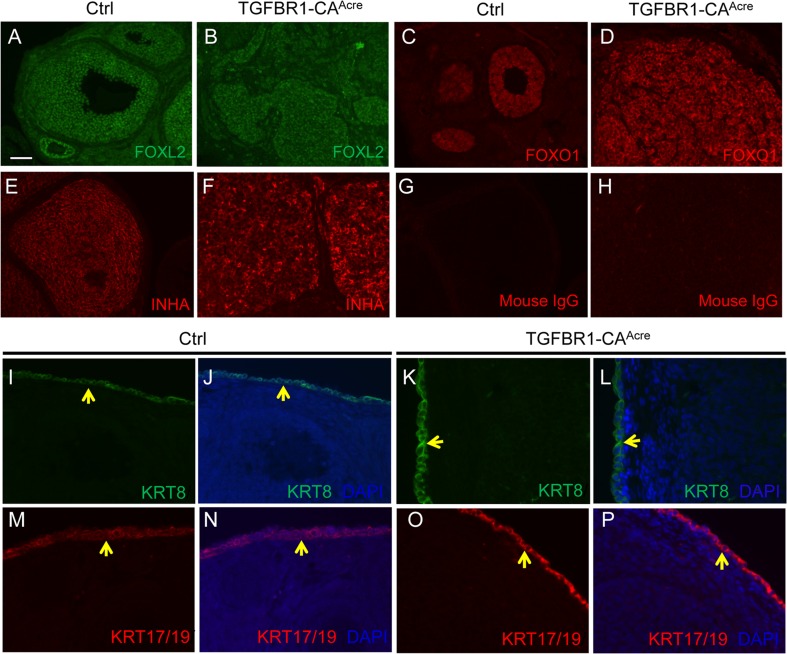
Molecular characterization of ovarian tumor type in TGFBR1-CA^**Acre**^ mice **A.**-**F.** Tumors in TGFBR1-CA^Acre^ mice express granulosa cell markers. Immunofluorescence of FOXL2 (A and B), FOXO1 (C and D), and INHA (E and F) in the control and TGFBR1-CA^Acre^ ovaries. Note the strong localization of all three proteins in the granulosa cells of control (A, C, and E) and tumor tissues in the TGFBR1-CA^Acre^ mice (B, D, and F). **G.** and **H.** Negative controls using isotype-matched mouse IgG. **I.**-**P.** Immunofluorescence of KRT8 and KRT17/19 in the control and TGFBR1-CA^Acre^ ovaries. Note that the expression of KRT8 (green; I-L) and KRT17/19 (red; M-P) was mainly detected in ovarian surface epithelia of both control and TGFBR1-CA^Acre^ ovaries (yellow arrows), but not in the tumor tissues (K, L, O, and P). DAPI was used to counterstain the nucleus. At least 4 control and TGFBR1-CA^Acre^ mice at the age of 2 months were analyzed by immunofluorescence and/or immunohistochemistry. Scale bar is representatively shown in (A) and equals 25 μm (I-P) and 50 μm (A-H).

To further define the molecular identity of gonadal tumors, we performed immunofluorescence to determine tumor cell expression of forkhead box L2 (FOXL2), a granulosa cell lineage marker [29], and three additional proteins that are abundantly expressed in granulosa cells, forkhead box O1 (FOXO1), INHA, and anti-Mullerian hormone (AMH). The expression of FOXL2 (Figure 2A), FOXO1 (Figure 2C), INHA (Figure 2E), and AMH (Figure S3A-C, G, and H) was confirmed in granulosa cells of control ovaries, with representative negative controls depicted (Figure 2G and 2H). We found that ovarian tumors from TGFBR1-CA^Acre^ mice were immunoreactive for FOXL2 (Figure 2B), FOXO1 (Figure 2D), and INHA (Figure 2F), supporting the development of granulosa cell tumors in these mice. However, expression of AMH was low in the tumor tissues (Figure S3D-F and I-K).

Because granulosa cells may transdifferentiate into Sertoli cells in granulosa cell tumors [12], we assessed whether there were Sertoli cell-like components in the TGFBR1-CA^Acre^ model by immunofluorescence using an antibody directed to SRY (sex determining region Y)-box 9 (SOX9), a Sertoli cell marker (Figure S4). While SOX9 was detected in some theca cells of control mice (Figure S4A-C) [30], aberrant SOX9 staining was found in TGFBR1-CA^Acre^ ovaries (Figure S4D-I). Expression of SOX9 in Sertoli cells was included as a positive control (Figure S4J and K).

**Figure 3:**
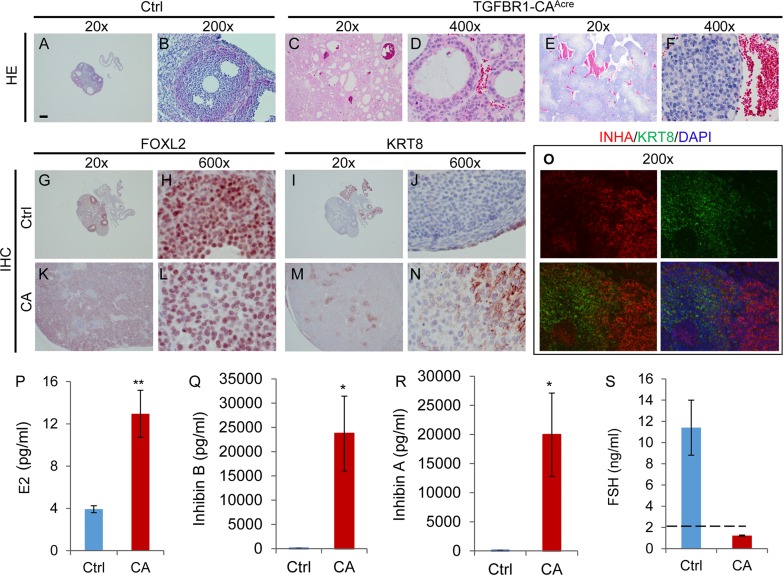
Histological, immunohistochemical, and hormonal analyses of ovarian tumors in TGFBR1-CA^**Acre**^ mice at late developmental stages **A.**-**F.** H&E staining of control and TGFBR1-CA^Acre^ ovaries demonstrates the arrangement of follicular (C and D; 7 month) and trabecular or cord-like (E and F; 6 month) patterns in tumors. Note the hemorrhage in the tumors *versus* controls (A and B; 7 month). *n* = 3 per group. **G.**-**N.** Immunohistochemical staining of FOXL2 and KRT8 in the control and TGFBR1-CA^Acre^ ovaries. Panels (H, L, J, and N) represent higher power images for panels (G, K, I, and M), respectively. Note the staining of FOXL2 in ovarian tumor tissues (K and L) and the expression of KRT8 in discrete cells (M and N) within TGFBR1-CA^Acre^ ovaries. **O.** Double immunofluorescence of KRT8 and INHA. Ovarian samples from control (*n* = 3) and TGFBR1-CA^Acre^ mice (*n* = 3) at the age of 6-7 months were analyzed by immunofluorescence and/or immunohistochemistry. Scale bar is representatively depicted in (A) and equals 10 μm (H, J, L, and N), 15 μm (D and F), 30 μm (B and O) and 300 μm (A, C, E, G, I, K, and M). **P.**-**S.** Hormone levels of E2, inhibin B, inhibin A, and FSH in the serum of control and TGFBR1-CA^Acre^ mice at the age of 5-7 months. Data are mean ± s.e.m. *n* = 7-8. ^*^P < 0.05 and ^**^P < 0.01.

Since *Amhr2*-Cre is also expressed in the ovarian surface epithelium [31], immunofluorescence was performed using antibodies against epithelial cell markers, cytokeratin 8 (KRT8) and 17/19 (KRT17/19), to exclude the epithelial cell identity of the neoplasms. As expected, KRT8 and KRT17/19 were localized to ovarian surface epithelia of control and TGFBR1-CA^Acre^ mice, with low to undetectable expression in the tumor tissues at the age of 2 months (Figure 2I–2P). Expression of KRT8 has been shown to be increased in mouse granulosa cell tumors [12]. We therefore further examined KRT8 expression during late stages of tumor development in our model. In contrast to the highly organized follicle structures in control mice (Figure 3A and 3B), TGFBR1-CA^Acre^ ovaries showed pathological changes such as the arrangement of follicular (Figure 3C and 3D) and/or trabecular (Figure 3E and 3F) patterns, the presence of hemorrhage (Figure 3C–3F), and multifocal necrosis within the center of some lobules (Figure S5A and B). The tumors were positive for FOXL2 (Figure 3K and 3L), which was expressed in control granulosa cells (Figure 3G and 3H). KRT8 was predominantly expressed in ovarian surface epithelia of controls (Figure 3I and 3J). However, a focal distribution of KRT8 in TGFBR1-CA^Acre^ ovaries was evident (Figure 3M and 3N). Double immunofluorescence revealed low expression or absence of INHA in KRT8-positive cells within tumor lobules (Figure 3O), suggesting distinct identity of KRT-positive cells.

### Hormone profile of mice harboring a constitutively active TGFBR1 in the ovary

Clinically, granulosa cell tumors often secrete excessive inhibin and estrogen [32, 33]. Serum inhibin is a reliable marker for granulosa cell tumors [34]. To determine the relevance of TGFBR1-CA^Acre^ mice as a potential model for granulosa cell tumors, we measured serum estradiol (E2), inhibin B, inhibin A, and follicle-stimulating hormone (FSH) levels in control and TGFBR1-CA^Acre^ mice. The results showed that serum levels of E2, inhibin B, and inhibin A were highly elevated in TGFBR1-CA^Acre^ mice *versus* controls (Figure 3P–3R). Consistent with the high levels of inhibin, serum FSH levels were below detection limit in TGFBR1 overactivated mice (Figure 3S). These data demonstrate that constitutive activation of TGFBR1 promotes the development of granulosa cell neoplasms which phenocopy certain molecular and hormonal characteristics of human granulosa cell tumors.

### Development of complementary mouse models by constitutive activation of TGFBR1 using additional ovary-expressed Cre lines

**Figure 4:**
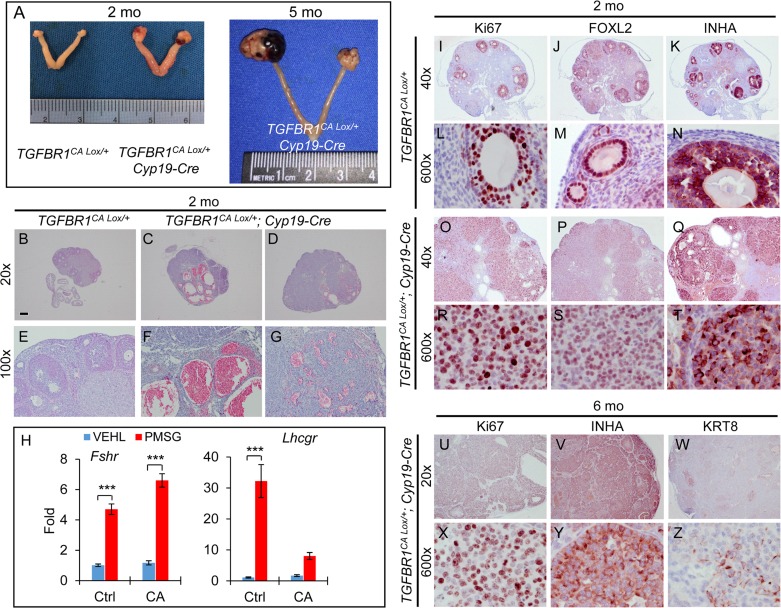
Constitutive activation of TGFBR1 in mouse granulosa cells using *Cyp19-*Cre promotes gonadal tumor formation **A.** Representative macroscopic images of ovarian tumors in *TGFBR1*CA ^Lox/+^; *Cyp19*-Cre mice. Macroscopic tumors were observed in *TGFBR1*CA ^Lox/+^; *Cyp19*-Cre mice (*n* = 22) examined at the age of 2-6 months. **B.**-**G.** H&E staining of ovaries from control (B and E; *n* = 4) and TGFBR1-CA^Ccre^ mice (C, D, F, and G; *n* = 3) at the age of 2 months. Panels (E, F, and G) represent higher power images for panels (B, C, and D), respectively. (C) and (D) represent the two ovaries from a *TGFBR1*CA ^Lox/+^; *Cyp19*-Cre mouse. Note the formation of multiple hemorrhagic ovarian cysts in one ovary (C and F) and hemorrhagic tumor foci in the other (D and G). **H.** The effect of PMSG treatment on ovarian mRNA expression of *Fshr* and *Lhcgr*. VEHL, vehicle control. Data are mean ± s.e.m. *n* = 5. ^***^P<0.001; two-way ANOVA. **I**.-**T**. Immunostaining of Ki67, FOXL2, and INHA in control (I-N) and *TGFBR1*CA ^Lox/+^; *Cyp19*-Cre ovaries (O-T) of 2-month-old mice. *n* = 3. **U.**-**Z.** Immunostaining of Ki67, INHA, and KRT8 in *TGFBR1*CA ^Lox/+^; *Cyp19*-Cre ovaries during late tumor development. Panels (L-N), (R-T), and (X-Z) represent higher power images for panels (I-K), (O-Q), and (U-W), respectively. Images are representative of immunohistochemistry analyses performed using 4-6 month old control (*n* = 3) and *TGFBR1*CA ^Lox/+^; *Cyp19*-Cre mice (*n* = 6). Scale bar is representatively depicted in (B) and equals 10 μm (L-N, R-T, and X-Z), 60 μm (E-G), 150 μm (I-K and O-Q), and 300 μm (B-D and U-W).

*Amhr2*-Cre is expressed in both ovarian granulosa cells and theca cells [35, 36] (Figure S1D). To substantiate our findings and determine whether constitutive activation of TGFBR1 in the granulosa cells contributes to the ovarian tumor phenotype, we generated two complementary mouse models that expressed TGFBR1^CA^ under the control of cytochrome P450 family 19-Cre (*Cyp19*-Cre) using *Tg(CYP19A1-cre)1Jri* mice, restricting Cre activity to the granulosa cells of antral follicles (Figure S1E), particularly those at late follicular stages [37]. Both *TGFBR1*^CA Lox/+^; *Cyp19*-Cre and *TGFBR1*^CA Lox/Lox^; *Cyp19*-Cre mice (herein termed TGFBR1-CA^Ccre^) developed ovarian sex cord-stromal tumors, which morphologically phenocopied those in *Amhr2*-Cre mice (Figure 4A and Figure S6A). Histological analysis demonstrated the formation of hemorrhagic ovarian cysts and tumor foci (Figure 4B–4G and Figure S6K-N). Consistent with the late onset of *Cyp19*-Cre expression in the ovary [37], ovarian weight was not significantly different between *TGFBR1*^CA Lox/+^; *Cyp19*-Cre and control mice at 1 month of age. *TGFBR1*^CA Lox/+^; *Cyp19*-Cre ovaries contained morphologically identifiable follicles at 3 weeks of age (data not shown), allowing further assessment of the effect of gonadotropin hormones on the differentiation of granulosa cells. The results showed that although pregnant mare serum gonadotropin (PMSG) could induce the expression of FSH receptor (*Fshr*) in both control and TGFBR1-CA^Ccre^ ovaries, the induction of luteinizing hormone/choriogonadotropin receptor (*Lhcgr*) was substantially compromised in the TGFBR1-CA^Ccre^ ovaries (Figure 4H). Moreover, tumors from TGFBR1-CA^Ccre^ ovaries were highly proliferative (Figure 4O, 4R, 4U, 4X and Figure S6E, H, G, J, O, and P), expressed granulosa cell markers FOXL2 (Figure 4P and 4S) and INHA (Figure 4Q, 4T, 4V, and 4Y and Figure S6F, I, G, J, Q, and R), and were immunoreactive for KRT8 during tumor progression (Figure 4W and 4Z and Figure S6S and T). Immunostaining of Ki67, FOXL2, and INHA using 2-month-old control ovaries are representatively depicted (Figure 4I–4N and Figure S6B-D).

Results from the TGFBR1-CA^Ccre^ mice demonstrated that overactivation of TGFBR1 in granulosa cells led to ovarian tumor development. Unexpectedly, we found that constitutive activation of TGFBR1 using GLI-Kruppel family member GLI1 (*Gli1*)-Cre^ERT2^ (designated as TGFBR1-CA^Gcre^; Figure S7A) caused the development of INHA-positive ovarian tumors following tamoxifen injection (Figure S7B-H). Although signals of red fluorescent protein (RFP; a *Gli1*-Cre reporter) was mainly found in theca layers, *Gli1*-Cre activity was also observed in a subset of granulosa cells within some follicles (Figure S7I and J) and signals were detected in granulosa cells and tumor tissues using *TGFBR1* probe by RNAscope (Figure S7K-N) in tamoxifen-treated mice, suggesting potential overactivation of TGFBR1 in granulosa cell compartment.

### Constitutively active TGFBR1 alters ovarian cell proliferation, differentiation, and tumor microenvironment

**Figure 5:**
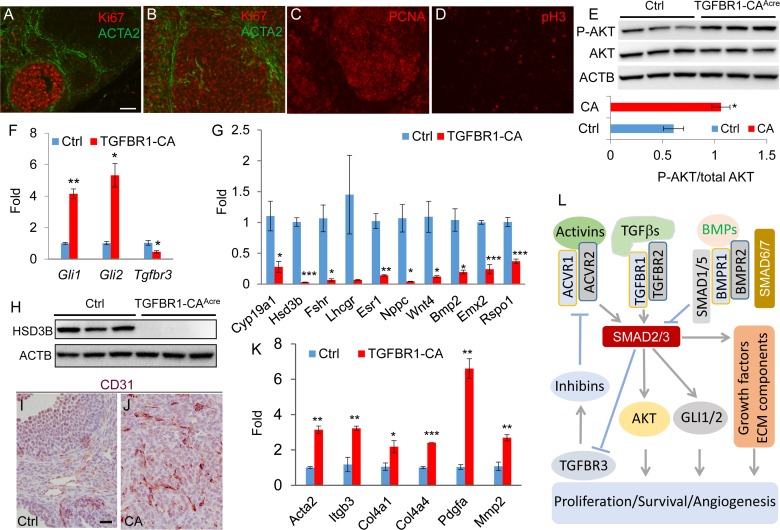
Constitutively active TGFBR1 alters ovarian cell proliferation, differentiation, and angiogenesis **A.**-**D.** Immunofluorescence of cell proliferation markers in 2-month-old control and/or TGFBR1-CA^Acre^ ovaries. ACTA2 (green) was utilized to mark the follicle structure. Scale bar is representatively depicted in (A) and equals 50 μm (A-D). **E.** Increased phospho-AKT levels in the ovaries of TGFBR1-CA^Acre^ mice by western blot using ovaries from 2-month-old mice. *n* = 3. Upper panels are representative western images and the lower bar graph is the quantification result. Each lane represents an independent sample. **F.** Increased *Gli1*/2 and reduced *Tgfbr3* mRNA abundance in the ovaries of TGFBR1-CA^Acre^ mice (2 month; n = 3) *versus* controls (*n* = 4). Data are mean ± s.e.m. ^*^P < 0.05 and ^**^P < 0.01. **G.** Dysregulation of genes associated with granulosa cell differentiation and function in the ovaries of 2-month-old TGFBR1-CA^Acre^ mice (*n* = 3) *versus* controls (*n* = 4). Data are mean ± s.e.m. ^*^P < 0.05, ^**^P < 0.01, and ^***^P < 0.001. **H.** Western blot analysis of HSD3B expression. n = 3. Each lane represents an independent sample. ACTB was used as an internal control. **I.** and **J.** Angiogenesis in ovarian tumors evidenced by immunohistochemical analysis of CD31 expression. Panels (I) and (J) represent the respective control and TGFBR1-CA^Acre^ ovaries of 2-month-old mice. *n* = 4. Scale bar is representatively depicted in (I) and equals 20 μm (I and J). **K.** Upregulation of genes associated with angiogenesis in 2-month-old TGFBR1-CA^Acre^ mice (*n* = 3) *versus* controls (*n* = 4). Data are mean ± s.e.m. ^*^P < 0.05, ^**^P < 0.01, and ^***^P < 0.001. **L.** Hypothetical model depicting potential TGFβ signaling targets (e.g, AKT, GLI1/2, and TGFBR3) during ovarian sex cord-stromal tumor development. TGFβ signaling activity is controlled by negative regulators (e.g., inhibitory SMADs and BMP signaling). Loss of repression of TGFβ and/or activin signaling by negative regulators promotes SMAD2/3 activation and alters cell proliferation and differentiation and angiogenesis.

An essential step for a normal cell to transform to a cancer cell is to acquire the ability of uncontrolled proliferation [38]. The highly proliferative nature and mitotic activity of tumor cells in the TGFBR1-CA^Acre^ ovaries were revealed by the presence of mitotic figures (Figure 1H), positive staining of cell proliferation markers Ki67 (Figure 5B) and proliferating cell nuclear antigen (PCNA; Figure 5C), and expression of mitotic marker phospho-histone H3 (pH3; Figure 5D). In control ovaries, Ki67 was strongly localized to proliferating granulosa cells (Figure 5A). Notably, theca cells positive for ACTA2 were highly organized in the control ovary (Figure 5A). However, ACTA2-positive cells were disoriented and/or enriched in the tumor foci (Figure 5B).

To determine how constitutive activation of TGFBR1 in the ovary tipped the cellular homeostatic scale in favor of proliferation, we examined the status of activation of AKT pathway, which promotes cell growth and survival. Western blot demonstrated increased levels of phospho-AKT in the TGFBR1-CA^Acre^ ovaries (Figure 5E). We also found that transcript levels of *Gli1* and *Gli2*, transcription factors that regulate the expression of target genes of hedgehog signaling, were highly elevated in TGFBR1 constitutively active ovaries (Figure 5F). Given the potential importance of GLI transcription factors in cancer development [6, 39], our findings suggest that TGFβ signaling may interact with hedgehog pathway to drive ovarian tumor formation [40–42]. In addition, the expression of mRNA transcripts encoding TGFBR3 (also known as betaglycan), a critical regulator of inhibin action [43], was reduced in the ovary of TGFBR1-CA^Acre^ mice compared with controls (Figure 5F).

Since coordinated cell proliferation and differentiation are critical for developmental events, we predicted that granulosa cell differentiation would be compromised in TGFBR1-CA^Acre^ mice. To test this, we analyzed a number of critical genes involved in granulosa cell differentiation and function, including *Cyp19a1*, 3-β-hydroxysteroid dehydrogenase (*Hsd3b*), *Fshr*, *Lhcgr*, estrogen receptor 1 (*Esr1*), natriuretic peptide type C (*Nppc*), *Wnt4*, *Bmp2*, empty spiracles homeobox 2 (*Emx2*), and R-spondin 1 (*Rspo1*). Our results revealed dysregulated expression of these genes in TGFBR1-CA^Acre^ ovaries (Figure 5G). Moreover, protein expression of HSD3B, an enzyme involved in progesterone biosynthesis, was also suppressed in the ovary of TGFBR1-CA^Acre^ mice (Figure 5H). These studies collectively suggest altered granulosa cell differentiation and function during ovarian tumor development.

Angiogenesis is a hallmark of cancer [38]. Consistent with the hemorrhagic tumor phenotype, active angiogenesis was found in these tumors, evidenced by immunostaining of platelet/endothelial cell adhesion molecule 1 (PECAM1)/CD31 (Figure 5I and 5J), an endothelial cell marker. Double immunofluorescence of CD31 and ACTA2 further revealed blood vessel formation within tumor foci (Figure S8). Furthermore, constitutive activation of TGFBR1 in the TGFBR1-CA^Acre^ ovaries caused increased expression of mRNA transcripts for *Acta2*, platelet-derived growth factor alpha (*Pdgfa*), integrin beta 3 (*Itgb3*), and extracellular matrix components including collagen type IV, alpha 1 and 4 (*Col4a1* and *Col4a4*) and matrix metalloproteinase 2 (*Mmp2*) (Figure 5K). Taken together, these data demonstrate that constitutively active TGFBR1 promotes cell proliferation, inhibits cell differentiation, and alters tumor microenvironment by enhancing angiogenesis. Based on our finding and existing literature, a hypothetical model is proposed to depict TGFβ signaling activation in gonadal tumor development (Figure 5L).

## DISCUSSION

Although *in vitro* studies have documented roles of TGFβ signaling in multifaceted cellular properties including proliferation and differentiation, the *in vivo* function of TGFβ signaling in the ovary is poorly defined. This knowledge gap is partially due to technical limitations of *in vitro* analysis, particularly cell-based assays, to fully address the function of TGFβ signaling from the tissue to the organismic level, where the biological response of a cell to a given TGFβ signal is contextually dependent and influenced by the availability of compensatory mechanisms [44].

TGFβ proteins inhibit cell proliferation, particularly epithelial cells, during early tumor development, but promote advanced tumor progression [45, 46]. However, transgenic overexpression of TGFβ1 in the skin appears to stimulate the growth of quiescent epidermal cells [47]. In addition, haploinsufficiency of *Tgfbr1* reduces the development of Kirsten rat sarcoma viral oncogene homolog (*KRAS*)*-*driven pancreatic precancer formation, uncovering a potential tumor promoting effect of TGFβ signaling [45, 48]. On the other hand, TGFβ stimulates the growth of cells of mesenchymal origin [45]. Notably, loss of BMP signaling receptors (i.e., BMPR1A/BMPR1B) and downstream SMADs (i.e., SMAD1/5/8) leads to gonadal tumor development and increased expression of TGFβ target genes and/or activation of SMAD2/3 [5, 6]. A recent study using *Smad1/5/4* triple conditional knockout mice provides further circumstantial evidence that SMAD4-mediated TGFβ/activin signaling might be tumorigenic in the ovary [49].

Based on the above evidence, we postulated that overactivation of TGFβ signaling in the ovary would promote tumorigenesis. To test this hypothesis, we created a mouse model harboring a constitutively active TGFBR1 that was conditionally activated by Cre recombinase driven by *Amhr2* promoter [5, 6]. The rapid and fully penetrant tumor phenotype clearly demonstrated an oncogenic effect of TGFβ signaling activation in the ovary. To our knowledge, this is the first mouse model for ovarian tumor development that is driven by constitutively active TGFBR1, without the dependence on additional oncogenic events such as KRAS activation [31, 45] or Neu overexpression [50]. Besides the ovary, we also observed myometrial abnormality in the TGFBR1-CA^Acre^ mice (unpublished observation), which is in line with our recent report demonstrating that sustained activation of TGFBR1 using progesterone receptor Cre alters uterine morphology [24]. The expression of *Amhr2*-Cre in several ovarian cell types [31, 35, 36] and its potential leaky feature [51] may represent a limitation of this mouse model. It is noteworthy that the tumor phenotype was not restricted to females, and males with constitutively active TGFBR1 (i.e., *TGFBR1*^CA Lox/+^; *Amhr2*-Cre) also developed testicular tumors (unpublished data that will be described in an independent report).

Generation of constitutively active TGFBR1 in the mouse ovary using *Cyp19*-Cre reproduced ovarian tumor phenotype, which corroborated findings from studies using *Amhr2*-Cre. It is interesting to note the focal expression of KRT8 in late-stage ovarian tumors in both TGFBR1-CA^Ccre^ and TGFBR1-CA^Acre^ mice. Since *Cyp19*-Cre is not expressed in the ovarian surface epithelium (Figure S1E) [31], our data suggest that overactivation of TGFBR1 in ovarian surface epithelial cells is not required for the observed KRT8 expression in tumor tissues. However, the possibility of the migration of ovarian surface epithelial cells into the tumor tissues cannot be excluded. Of note, only a limited number of *TGFBR1*^CA Lox/Lox^; *Cyp19*-Cre female mice were obtained during the course of the experiment because *TGFBR1*^CA Lox/+^; *Cyp19*-Cre male breeders were also prone to testicular tumor formation at an early reproductive age (Figure S9), presumably due to *Cyp19*-Cre expression in the testis [52].

A somewhat unexpected finding was that activation of TGFBR1 using inducible *Gli1*-Cre caused ovarian tumor formation. Although *Gli1* is known to be expressed in theca cells, there is evidence supporting the expression of *Gli1* mRNA in mouse granulosa cells [6, 53]. This could potentially explain the observation of ovarian tumor development resulting from TGFBR1^CA^ activation by *Gli1*-Cre. It is also possible that the Cre is leaky or that sustained activation of TGFBR1 in ovarian theca cells may confer a rapid GLI1-induced response in the granulosa cells, leading to Cre recombinase expression and subsequent activation of TGFBR1^CA^. The exact mechanism awaits further elucidation. Nevertheless, the complementary mouse models created by the current study, particularly the TGFBR1-CA^Ccre^ models, are valuable in future studies to define genes/pathways that are related to granulosa cell specific activation of TGFBR1 during ovarian tumor initiation and progression.

Our further effort toward understanding how constitutively active TGFBR1 in the ovary led to tumorigenesis generated the following insights. First, it has been shown that TGFβ signaling regulates phosphatidylinositol 3-kinase (PI3K)-AKT [54, 55], whose activation is widely implicated in cancer cell growth and survival [56] and associated with human cancer development [57]. TGFBR1 activation increases mammary epithelial cell survival and facilitates oncogene-induced malignant transformation *via* PI3K-AKT pathway [50]. Thus, it is conceivable that activation of AKT, as a direct or indirect consequence of TGFβ signaling activation, accelerates tumor progression in the TGFBR1-CA^Acre^ mice. Second, ovarian tumors in TGFBR1-CA^Acre^ mice had higher levels of *Gli1* and *Gli2* mRNAs. The expression of *Gli* genes that encode transcription factor effectors of sonic hedgehog signaling is also upregulated in the granulosa cell tumors of *Bmpr1a*/*Bmpr1b* conditional knockout mice [6]. Based on the interplay between Hedgehog and TGFβ signaling during ovarian carcinogenesis [6, 39], the involvement of TGFBR1-GLI1/2 signaling axis in granulosa cell tumor development seems plausible. Third, we found that *Tgfbr3* mRNA abundance was reduced in ovarian tissues with sustained activation of TGFBR1. TGFBR3 is a determinant of the inhibin action/potency [43] and inhibits the survival of human granulosa tumor cells [58]. Hence, reduced expression of TGFBR3 may facilitate the pathogenesis of ovarian tumors *via* attenuating inhibin function and potentiating activin signaling [59, 60].

Emerging evidence indicates that TGFβ signaling is activated in human granulosa cell tumors [12, 49, 61]. Of note, a recent study has shown that ~97% of adult granulosa cell tumors carry a *FOXL2* somatic missense mutation [62], highlighting a breakthrough in the field of granulosa cell tumor research. The fact that target genes of mutant FOXL2 in granulosa cells are enriched for TGFβ signaling [63] further supports the importance of TGFβ signaling in the pathogenesis of human granulosa cell tumors. Thus, future studies are needed to test the effect of TGFBR1 modulators on the cellular properties and the development of human granulosa cell tumors, as may yield novel insights into the treatment of these tumors.

An interesting question raised by this study is why gonads are prone to oncogenesis upon constitutive activation of TGFBR1. Cancer stem cells (CSCs), a potential source of cancer cells within a neoplasm, are capable of self-renewal. TGFβ signaling has been shown to promote the expression of CD133, a cancer stem cell marker, in hepatic epithelial cells, leading to tumor formation in the xenograft [64]. It is tempting to speculate that constitutively active TGFBR1 promotes the acquisition of the stemness of ovarian somatic cells to facilitate tumorigenesis. However, little is known about CSCs in ovarian sex cord-stromal tumors. It is also plausible, as described above, that sustained activation of TGFBR1 may alter inhibin/activin signaling activity, leading to gonadal tumorigenesis [4, 59, 60]. Further studies are needed to address these possibilities.

In summary, we provide genetic evidence for the oncogenic effect of TGFβ signaling activation in gonadal tumor development. In-depth analysis is expected to shed new light on the etiology of ovarian granulosa cell tumors. These new mouse models are potentially valuable for preclinical testing of targeted therapies to treat ovarian granulosa cell tumors.

## MATERIALS AND METHODS

### Animals

Animal manipulation is in accordance with the guidelines of the Institutional Animal Care and Use Committee (IACUC) at Texas A&M University. Protocols using laboratory mice for this study were approved by the IACUC. Mice were maintained on a C57BL/6; 129SvEv genetic background and had access to food and water ad libitum. The *Amhr2-*Cre (*Amhr2^tm3(cre)Bhr^*) mice were generated as described [65] and expression of *Amhr2*-Cre in ovarian somatic cells has been documented [5, 31, 36]. *Cyp19*-Cre [*Tg(CYP19A1-cre)1Jri*] is expressed in granulosa cells [66, 67]. Protocols using *Gli1*-Cre^ERT2^ mice were performed under the UK Home Office guidelines. The *Gli1*-Cre^ERT2^ mice [68] and mice harboring a Cre-inducible *LacZ* allele, *Gt(ROSA)26Sor^tm1Sor^/J* [27], were obtained from The Jackson Laboratory. The mice were on 129S6/SvEvTac background and crossed to C57BL/6J mice for at least four generations according to the supplier. Mice containing a latent constitutively active TGFBR1 were created as described [22].

### Mouse breeding, genotyping, fertility test, and treatment

To conditionally activate TGFBR1 in the ovary, mice containing *TGFBR1*^CA^ were crossed with *Amhr2*-Cre or *Cyp19*-Cre mice. The resultant mice were termed TGFBR1-CA^Acre^ and TGFBR1-CA^Ccre^, respectively. Genotyping and/or DNA recombination analysis of *TGFBR1*^CA^ [22, 23], *Amhr2*-Cre [69], and *Cyp19*-Cre [67] were conducted using PCR. The age of mice was reported as a rounded value when it is not an integer for week/month. For the fertility test, TGFBR1-CA^Acre^ females and controls were caged with proven fertile males at the age of 6-8 weeks for a period of 3 months. To determine ovarian response to gonadotropin, TGFBR1-CA^Ccre^ and control mice were treated with PMSG (5U) or VEHL intraperitoneally (i.p.). Ovarian samples were collected for RNA isolation 48 h post PMSG treatment. To activate *TGFBR1*^CA^ in the *Gli1*-positive cell population, *TGFBR1*^CA^ mice were crossed with mice expressing an inducible Cre under the control of *Gli1* promoter [68]. Mice harboring both *Gli1-*Cre and *TGFBR1*^CA^ were designated as TGFBR1-CA^Gcre^. To induce Cre expression, 6-8 week-old TGFBR1-CA^Gcre^ mice were injected (i.p.) daily for 4 days, with 3 mg tamoxifen (Sigma) on day 1, and 2 mg on days 2, 3, and 4. Mice were sampled 4 days after induction for *in situ TGFBR1* mRNA expression analysis (RNAscope) or when clinical signs of tumors developed. RFP expression experiment was performed 7 days after mice carrying both *Gli1-*Cre^ERT2^ and Lox-Stop-Lox (LSL)-RFP reporter were treated with tamoxifen [70].

### Histological analysis

Ovarian samples were collected from control and experimental mice and processed for histological analysis. A standard protocol was used for hematoxylin and eosin (H&E) staining. Microscopic images were captured using DP25 (Olympus) digital camera interfaced with cellSens Imaging Software.

### Immunofluorescence microscopy and immunohistochemistry

Immunofluorescence and immunohistochemistry were performed using serial paraffin sections (5 μm) [18], with primary antibodies listed in Table S1. Secondary antibodies for immunofluorescence were conjugated with Alexa Fluor 488 or 594. The sections were mounted using ProLong Gold Slowfade media containing 4′,6-diamidino-2-phenylindole (DAPI; Invitrogen). Images were captured using IX73 microscope (Olympus) interfaced with an XM10 CCD camera and cellSens Software. Immunohistochemistry was performed using avidin-biotin complex (ABC) method as described [18]. Signals were developed using VECTOR NovaRED. Negative controls where primary antibodies were replaced by isotype-matched IgGs from the same species were included.

### RNAscope

Chromogenic RNAscope (Advanced Cell Diagnostics, Hayward, CA) was performed on formalin-fixed paraffin-embedded sections using *TGFBR1* probe (Hs-TGFBR1, Cat no. 431041) accordingly to the manufacturer instructions.

### Western blot

Protein samples were prepared and quantified as described [19]. Approximately 30 μg of proteins were resolved on 12% Mini-PROTEAN TGX Precast Gels (Bio-Rad) and then transferred to polyvinylidene difluoride (PVDF) membranes (Bio-Rad). The membranes were incubated with primary antibodies (Table S1) at 4°C overnight, followed by incubation with horseradish peroxidase (HRP)-conjugated donkey anti-rabbit antibody or anti-goat antibody (1:20,000 in 5% milk; Jackson ImmunoResearch) at room temperature for 1 h. Immobilon Western Chemiluminescent HRP Substrate (Millipore) was used to develop the signals. Blots were scanned using a Kodak Image Station 4000 mm PRO. Beta actin (ACTB) was included as an internal control to normalize potential variations among protein samples. Quantification of western blot was performed using ImageJ (NIH, 1.47v).

### Hormone analyses

Serum E2, inhibin B and A, and FSH levels were measured using the Ligand Assay and Analysis Core at the Center for Research in Reproduction, University of Virginia. Assay details can be found at https://med.virginia.edu/research-in-reproduction/laboratory-facilities/assay-methods/.

### X-gal staining

Ovarian samples from mice harboring *Gt(ROSA)26Sor^tm1Sor^/J*; *Amhr2*-Cre or *Gt(ROSA)26Sor^tm1Sor^/J; Cyp19*-Cre were fixed in 2% paraformaldehyde and 0.2% glutaraldehyde (pH 7.4) for 15 min at 4°C. The samples were washed and then stained using 1 mg/ml X-gal solution containing 5 mM potassium ferricyanide and 5 mM potassium ferrocyanide. After staining, the ovaries were processed for post-fixation, paraffin embedding, sectioning, and counter staining using fast red [18].

### RNA isolation, conventional PCR, and real-time PCR

Total RNA from mouse ovaries was isolated using RNeasy Mini Kit (Qiagen). Reverse transcription of complementary DNA (cDNA) was then conducted as described [18]. For conventional PCR, *TGFBR1*^CA^ was amplified from ovarian cDNA using oligo primers (Forward: 5′-TTGTGAACAGAAGTTAAGGC-3′; Reverse: 5′-AGCATAATCAGGAACATCAT-3′) [22]. PCR products were separated using 1% agarose gel containing ethidium bromide. Real-time RT-PCR (qPCR) was performed on a CFX Connect Real-time PCR Detection System (Bio-Rad) using iTaq Universal SYBR Green Supermix (Bio-Rad), cDNA, and gene specific primers (Table S2) in a total volume of 10 μl. The PCR conditions were described elsewhere [69]. Amplification of ribosomal protein L19 (*Rpl19*) was included as an internal control, and calculation of relative levels of gene expression was based on DDCT method [71].

### Statistical analyses

All statistical analyses were performed using IBM Statistical Package for the Social Sciences (SPSS; Version 23) except the survival rate analysis by Mantel-Cox test using GraphPad Prism 6 software. Two sample independent *t*-test (unpaired) was performed to determine difference between two means. The homogeneity of variance was assessed using Levene's test. Two-way analysis of variance (ANOVA) was applied to determine the effect of gonadotropin on granulosa cell differentiation. Data are presented as mean ± standard error of the mean (s.e.m) unless otherwise specified. Statistical significance was defined at *P* < 0.05. Results are marked as ^*^P < 0.05, ^**^P < 0.01, and ^***^P < 0.001.

## SUPPLEMENTARY MATERIALS FIGURES AND TABLES


