# Supplementary material for: Constitutively active transforming growth factor β receptor 1 in the mouse ovary promotes tumorigenesis
Source: Oncotarget. 2016 Jun 17;7(27):40904–18. doi: 10.18632/oncotarget.10149 (PMC5173031; doi:10.18632/oncotarget.10149)
Supplement: Supplementary file 1 [file oncotarget-07-40904-s001.pdf]

Constitutively active transforming growth factor β receptor 1 in the mouse ovary promotes tumorigenesis

Supplementary Material

Table S1: Primary antibodies for immunohistochemistry, immunofluorescence, and western blot.

Name	Source	Host	IF/IHC	Western
ACTA2	Abcam	Mouse	1:2000	
MSY2	Abcam	Rabbit	1:200	
HA	Santa Cruz	Rabbit		1:200
RFP	Abcam	Rabbit	1:200	
Phospho-SMAD2	Millipore	Rabbit		1:500
SMAD2	Cell signaling	Rabbit		1:1000
ACTB	Sigma	Mouse		1:50000
Ki67	Abcam	Rabbit	1:200	
PCNA	Cell Signaling	Mouse	1:5000	
Phospho-histone H3	Cell Signaling	Rabbit	1:200	
HSD3B	Santa Cruz	Goat		1:1000
SOX9	Millipore	Rabbit	1:1000	
INHA	AbD Serotec	Mouse	1:100	
FOXL2	Abcam	Goat	1:1500	
FOXO1	Cell Signaling	Rabbit	1:400	
AMH	Santa Cruz	Goat	1:1000	
KRT8	Developmental Studies Hybridoma Bank	Rat	1:100	
KRT17/19	Cell Signaling	Rabbit	1:300	
PECAM1/CD31	Abcam	Rabbit	1:200	
Phospho-AKT	Cell Signaling	Rabbit		1:2000
AKT	Cell Signaling	Rabbit		1:1000

Table S2. Primers for quantitative real-time PCR

Name	Sequence (5′-3′)		Reference
Cyp19a1	Forward	TGGACGAAAGTGCTATTGTGAA	[1]
	Reverse	TCTTTCAAGTCCTTGACGGAT	
Hsd3b	Forward	TCCGACCAGAAACCAAGG	[2]
	Reverse	GCACTGGGCATCCAGAAT	
Fshr	Forward	CCTTGCTCCTGGTCTCCTTG	PrimerBank ID 31980789a1
	Reverse	CTCGGTCACCTTGCTATCTTG	
Lhcgr	Forward	GAAATGGATTTGAAGAAGTACAAAG	[2]
	Reverse	CCATTGTGCATCTTCTCCAG	
Esr1	Forward	TTGTGTGCCTCAAATCCATC	
	Reverse	GAGATGCTCCATGCCTTTGT	
Tgfbi	Forward	CGCCAAGTCACCCTACCAG	[3]
	Reverse	TGCACAGCACATACATTGGGG	
Amh	Forward	CCACACCTCTCTCCACTGGTA	PrimerBank ID 6680686a1
	Reverse	GGCACAAAGGTTCAGGGGG	
Smad7	Forward	GGGCTTTCAGATTCCCAACTT	[3]
	Reverse	CACGCGAGTCTTCTCCTCC	
Serpine1	Forward	TTCAGCCCTTGCTTGCCTC	[4]
	Reverse	ACACTTTTACTCCGAAGTCGGT	
Ctgf	Forward	GGGCCTCTTCTGCGATTTC	[4]
	Reverse	ATCCAGGCAAGTGCATTGGTA	
Acta2	Forward	GTCCCAGACATCAGGGAGTAA	[5]
	Reverse	TCGGATACTTCAGCGTCAGGA	
Itgb3	Forward	CCACACGAGGCGTGAACTC	PrimerBank ID 7949057a1
	Reverse	CTTCAGGTTACATCGGGGTGA	
Gli1	Forward	CCAAGCCAACTTTATGTCAGGG	PrimerBank ID 6754002a1
	Reverse	AGCCCGCTTCTTTGTTAATTTGA	
Gli2	Forward	CAACGCCTACTCTCCCAGAC	PrimerBank ID 21411092a1
	Reverse	GAGCCTTGATGTACTGTACCAC	
Tgfbr3	Forward	GGTGTGAACTGTCACCGATCA	PrimerBank ID 33469109a1
	Reverse	GTTTAGGATGTGAACCTCCCTTG	
Col4a1	Forward	CTGGCACAAAAGGGACGAG	[5]
	Reverse	ACGTGGCCGAGAATTTCACC	
Col4a4	Forward	ATGAGGTGCTTTTTCAGATGGAC	[5]
	Reverse	GGGGCCGCCATACTTCTTG	
Pdgfa	Forward	GAGGAAGCCGAGATACCCC	[5]
	Reverse	TGCTGTGGATCTGACTTCGAG	
Nppc	Forward	CAGAAAAAGGGTGACAAGACTCC	PrimerBank ID 6754880a1
	Reverse	ATCCCAGACCGCTCATGGA	
Wnt4	Forward	CATCGAGGAGTGCCAATACCA	[6]
	Reverse	GGAGGGAGTCCAGTGTGGAA	
Bmp2	Forward	GGGACCCGCTGTCTTCTAGT	[4]
	Reverse	TCAACTCAAATTCGCTGAGGAC	
Emx2	Forward	GTCCCAGCTTTTAAGGCTAGA	[7]
	Reverse	CTTTTGCCTTTTGAATTTCGTTC	
Rspo1	Forward	GGGATCAAGGGCAAGAGACAG	PrimerBank ID 20149776a1
	Reverse	CTGGCGGATGTCGTTCCTC	
Mmp2	Forward	CAAGTTCCCCGGCGATGTC	PrimerBank ID 6678902a1
	Reverse	TTCTGGTCAAGGTCACCTGTC	
Rpl19	Forward	ATGAGTATGCTCAGGCTACAGA	[5]
	Reverse	GCATTGGCGATTTCATTGGTC	

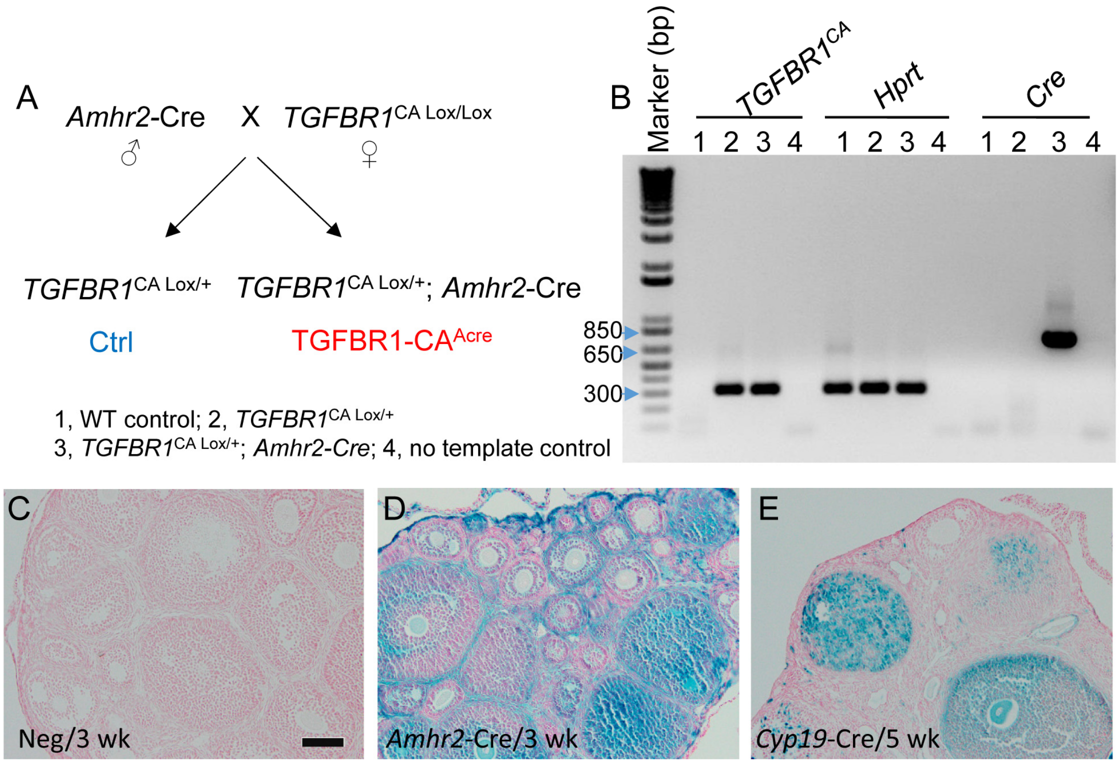

Figure S1. Generation of mice containing a constitutively active TGFBR1 in the ovary. (**A**) Breeding strategy. The *TGFBR1*$^{\text{CA Lox/Lox}}$ mice were crossed with mice harboring *Amhr2*-Cre to generate controls (*TGFBR1*$^{\text{CA Lox/+}}$; Ctrl) and experimental mice containing a constitutively active *TGFBR1* in the ovary (*TGFBR1*$^{\text{CA Lox/+}}$; *Amhr2*-Cre; TGFBR1-CA^{Acre}). (**B**) Representative genotyping PCR. The *TGFBR1*^{CA}, *Hprt*, and Cre were detected using specific primers. Lanes 1-4 represent WT, *TGFBR1*$^{\text{CA Lox/+}}$, and *TGFBR1*$^{\text{CA Lox/+}}$; *Amhr2*-Cre, and negative controls with no DNA template, respectively. (**C-E**) X-gal staining of ovaries from *ROSA26*; *Amhr2*-Cre (n = 7) and *ROSA26*; *Cyp19*-Cre mice (n = 3). Mice that did not carry Cre were used as negative controls (n = 3). Note that the blue staining represents *Amhr2*-Cre (D) and *Cyp19*-Cre (E) activity in the ovary. The control ovary showed no specific staining (C). The sections were counterstained with fast red. Scale bar is representatively shown in (C) and equals 100 µm (C-E).

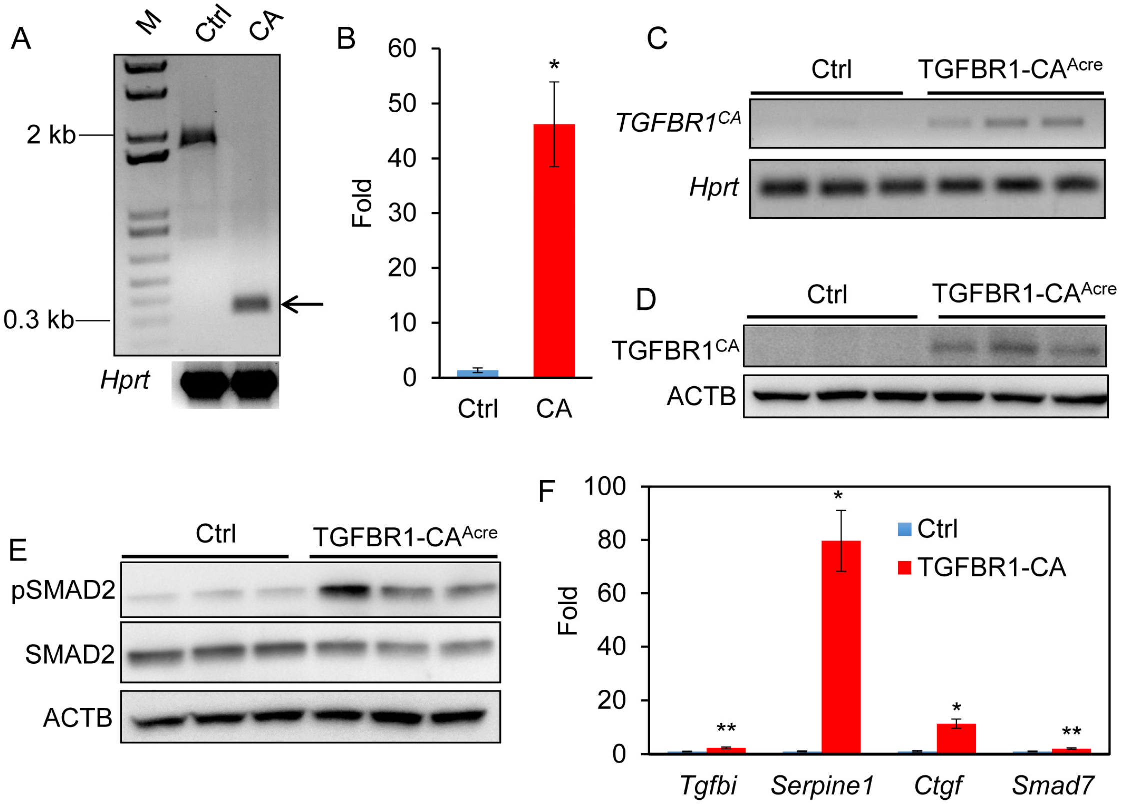

Figure S2. Validation of constitutive activation of TGFBR1 in the mouse ovary. (**A**) DNA recombination. Recombined *TGFBR1*^{CA} was detected by PCR in the TGFBR1-CA^{Acre} ovary (Arrow). The ~2 kb band in Ctrl represents non-recombined allele. M, DNA marker. (**B-D**) Detection of *TGFBR1*^{CA} mRNA transcripts and proteins using quantitative real-time PCR (B; n = 4 for Ctrl and n = 3 for TGFBR1-CA^{Acre}), conventional PCR (C; n = 3), and western blot (D; n = 3). Note the high levels of *TGFBR1*^{CA} mRNA and fusion proteins in the TGFBR1-CA^{Acre} ovaries versus controls. TGFBR1^{CA} fusion proteins were detected using an anti-HA antibody. Data are mean ± s.e.m. $^{*}P < 0.05$. (**E**) Western blot analysis of phospho-SMAD2 in the ovaries of TGFBR1-CA^{Acre} and controls. Note that increased levels of phospho-SMAD2 were detected in TGFBR1-CA^{Acre} ovaries (n = 3) versus controls (n = 3). ACTB was included as an internal control. Each lane in panels (C-E) represents an independent sample. (**F**) Expression of mRNAs for TGFβ target genes in the ovaries of mice at the age of 2 months. Note the induction of *Tgfbi*, *Serpine1*, *Ctgf*, and *Smad7* in the TGFBR1-CA^{Acre} ovaries (n = 3) compared with controls (n = 4). Data are mean ± s.e.m. $^{*}P < 0.05$; $^{**}P < 0.01$.

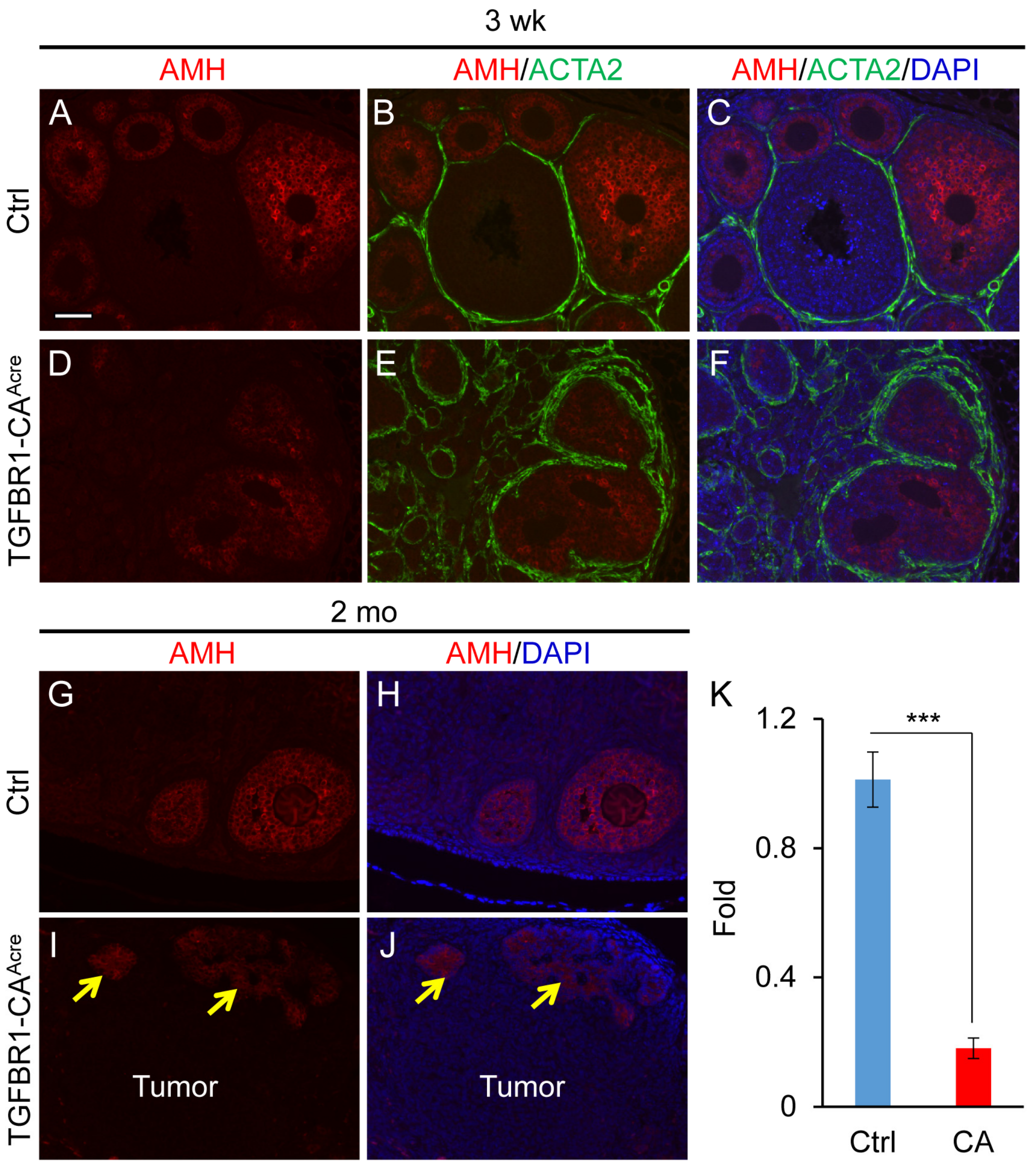

Figure S3. Altered expression of AMH in the ovary of TGFBR1-CA^{Acre} mice. (**A-F**) Double immunofluorescence of AMH (red) and ACTA2 (green) in the control (A-C) and TGFBR1-CA^{Acre} mice (D-F) at the age of 3 wks. ACTA2 marked the theca layer of follicles in the controls (B and C). AMH signals were detected in preantral and small antral follicles. In the TGFBR1-CA^{Acre} ovary, theca layers were disorganized and AMH expression was low. (**G-J**) immunofluorescence of AMH (red) in control and TGFBR1-CA^{Acre} ovaries at the age of 2 months. Note the absence of AMH signals in the tumor foci and the presence of disorganized follicles nearby that expressed AMH (yellow arrows). DAPI was used to counterstain the nucleus. Ovaries from 6 mice (n = 3 per group) were analyzed by immunofluorescence. Scale bar is representatively shown in (A) and equals 50 µm (A-J). (**K**) Reduced *Amh* mRNA expression during ovarian tumor development. n = 3-4. Data are mean ± s.e.m. $^{*}P < 0.001$.

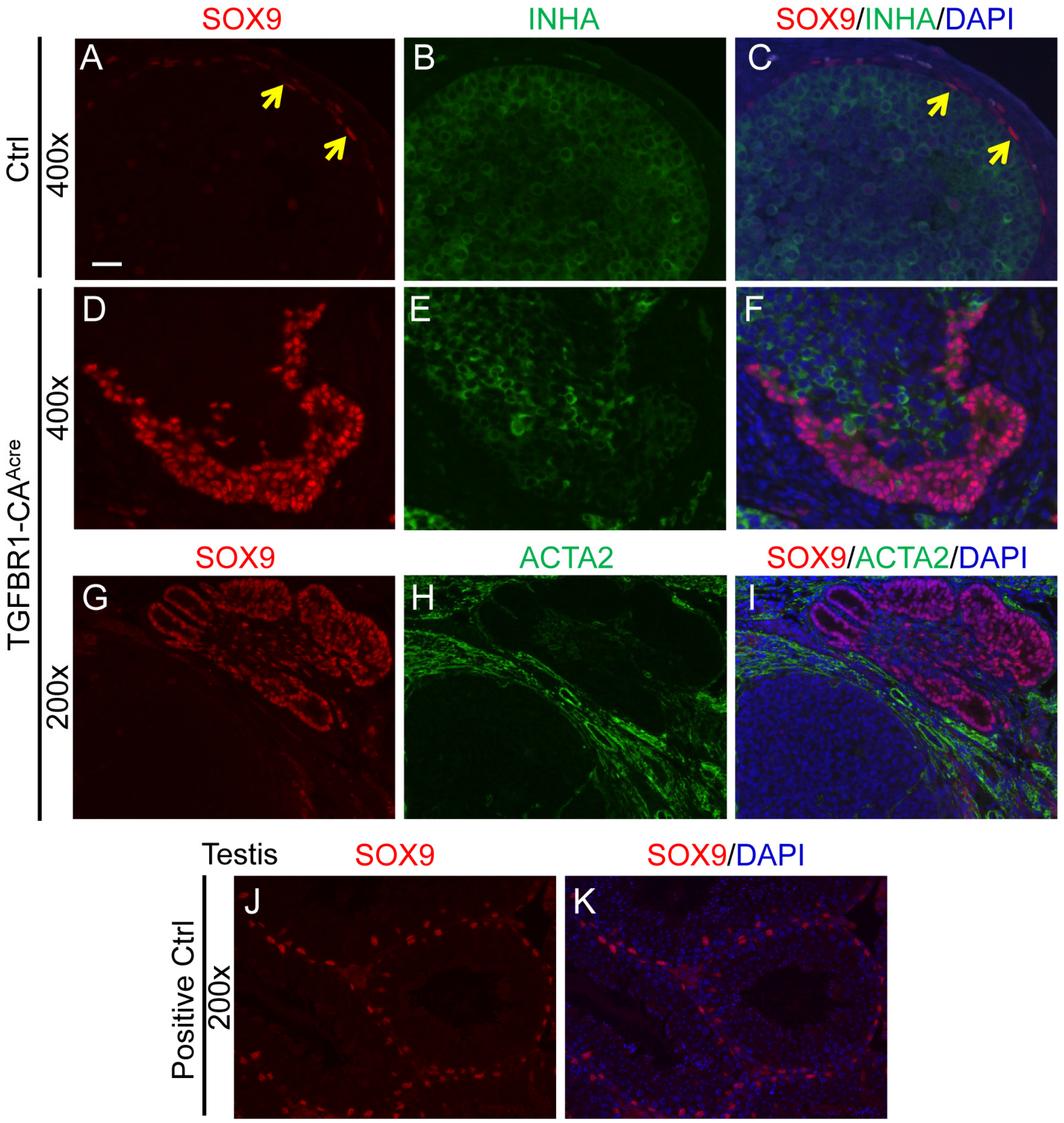

Figure S4. Localization of SOX9 to the ovaries of control and TGFBR1-CA^{Acre} mice. (**A-F**) Double immunofluorescence of SOX9 (red) and INHA (green) in the ovaries of control (A-C) and TGFBR1-CA^{Acre} mice (D-F). Note that the staining of SOX9 was confined to the theca layers in the controls (A and C; yellow arrows), but distributed in a localized pattern (D and F) within the ovary of TGFBR1-CA^{Acre} mice. SOX9-positive cells expressed low to undetectable levels of INHA (F). (**G-I**) Double immunofluorescence of SOX9 (red) and ACTA2 (green) in the ovary of TGFBR1-CA^{Acre} mice. Note that SOX9 was localized to cord-like structures. Images are representative of immunofluorescence microscopy performed using control (n = 2) and TGFBR1-CA^{Acre} mice (n = 3) at the age of 2 months. (**J** and **K**) Positive controls for SOX9 staining using the testis from adult wild-type mice. SOX9 (red) was expressed in the Sertoli cells of the testis. DAPI was used to counterstain the nucleus. Scale bar is representatively shown in (A) and equals 20 μm (A-F) and 40 μm (G-K).

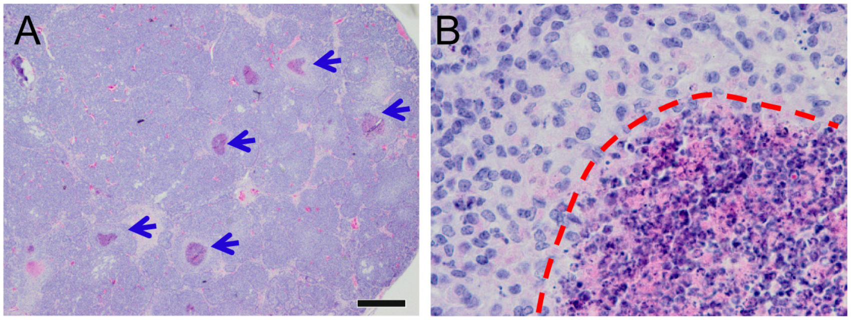

Figure S5. Necrosis in ovarian tumors from TGFBR1-CA^{Acre} mice. (**A** and **B**) H&E staining. Note the presence of multifocal necrosis and loss of neoplastic cells within the center of multiple tumor foci. Panel (B) represents a higher power image for (A). Blue arrows indicate necrosis, and dashed line demarcates necrotic region. Ovaries from 6 mice (n = 3 per group) at the age of 6-7 months were stained with H&E. Scale bar is representatively shown in (A) and equals 25 µm (B) and 500 µm (A).

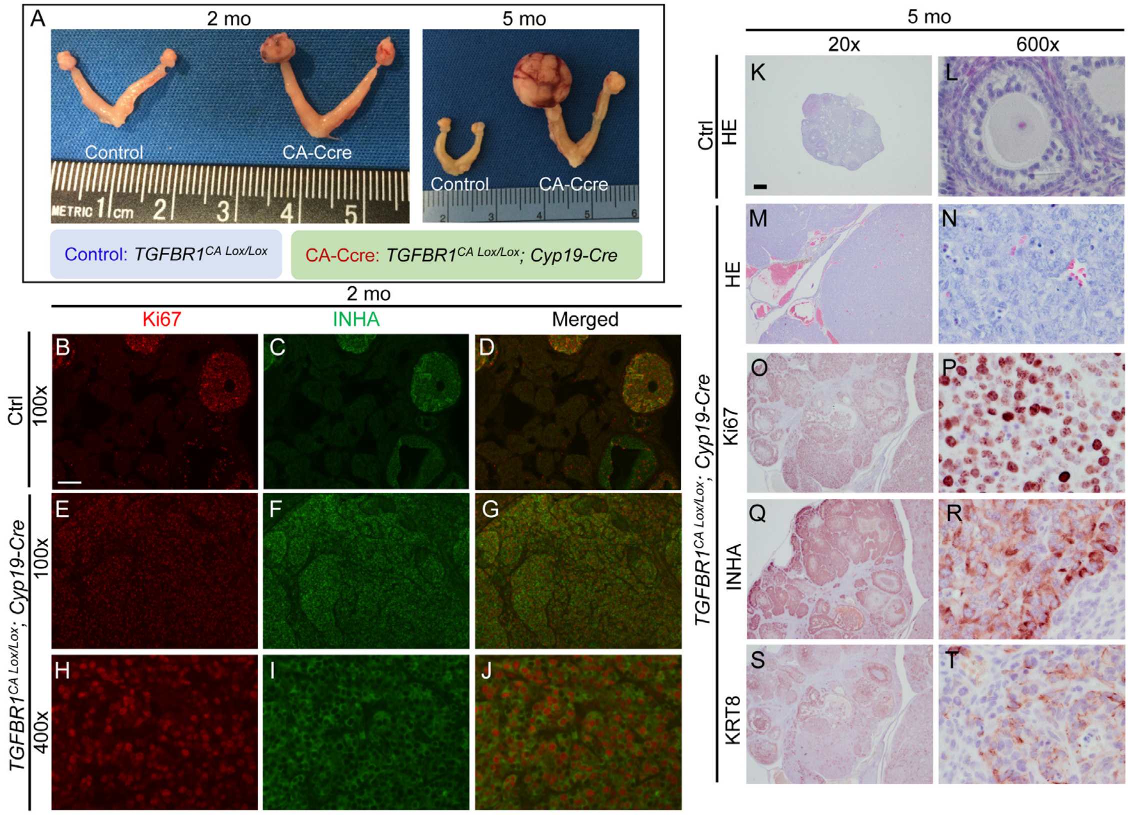

Figure S6. *TGFBR1^{CA}* $^{\text{Lox/Lox}}$; *Cyp19*-Cre mice develop sex-cord stromal tumors. (**A**) Representative macroscopic images of ovarian tumors in *TGFBR1^{CA}* $^{\text{Lox/Lox}}$; *Cyp19*-Cre mice at the age of 2-5 months (n = 14). (**B-J**) Double immunofluorescence of Ki67 and INHA using ovaries of control (B-D) and *TGFBR1^{CA}* $^{\text{Lox/Lox}}$; *Cyp19*-Cre mice (E-J) at the age of 2 months. Panels (H, I, and J) represent higher power images for panels (E, F, and G), respectively. Ki67 (red) and INHA (green)-positive cells were found across the entire ovarian tissue section of the *TGFBR1^{CA}* $^{\text{Lox/Lox}}$; *Cyp19*-Cre mice. Ovaries from 8 mice (n = 4 per group) at the age of 2-3 months were analyzed using immunofluorescence and/or immunohistochemistry. Scale bar is representatively depicted in (B) and equals 100 μm (B-G) and 25 μm (H-J). (**K-N**) H&E staining of ovaries from control (K and L) and *TGFBR1^{CA}* $^{\text{Lox/Lox}}$; *Cyp19*-Cre mice (M and N) at 5 months of age. Panels (L and N) represent higher power images for panels (K and M), respectively. H&E staining was performed using 5 control and 6 *TGFBR1^{CA}* $^{\text{Lox/Lox}}$; *Cyp19*-Cre mice. (**O-T**) Immunostaining of Ki67, INHA, and KRT8 using ovaries from *TGFBR1^{CA}* $^{\text{Lox/Lox}}$; *Cyp19*-Cre mice. Panels (P, R, and T) represent higher power images for panels (O, Q, and S), respectively. Immunohistochemistry analysis was performed using control (n = 5) and *TGFBR1^{CA}* $^{\text{Lox/Lox}}$; *Cyp19*-Cre mice (n = 5) at the age of 5 months. Scale bar is representatively shown in (K) and equals 300 μm (K, M, O, Q, and S) and 10 μm (L, N, P, R, and T).

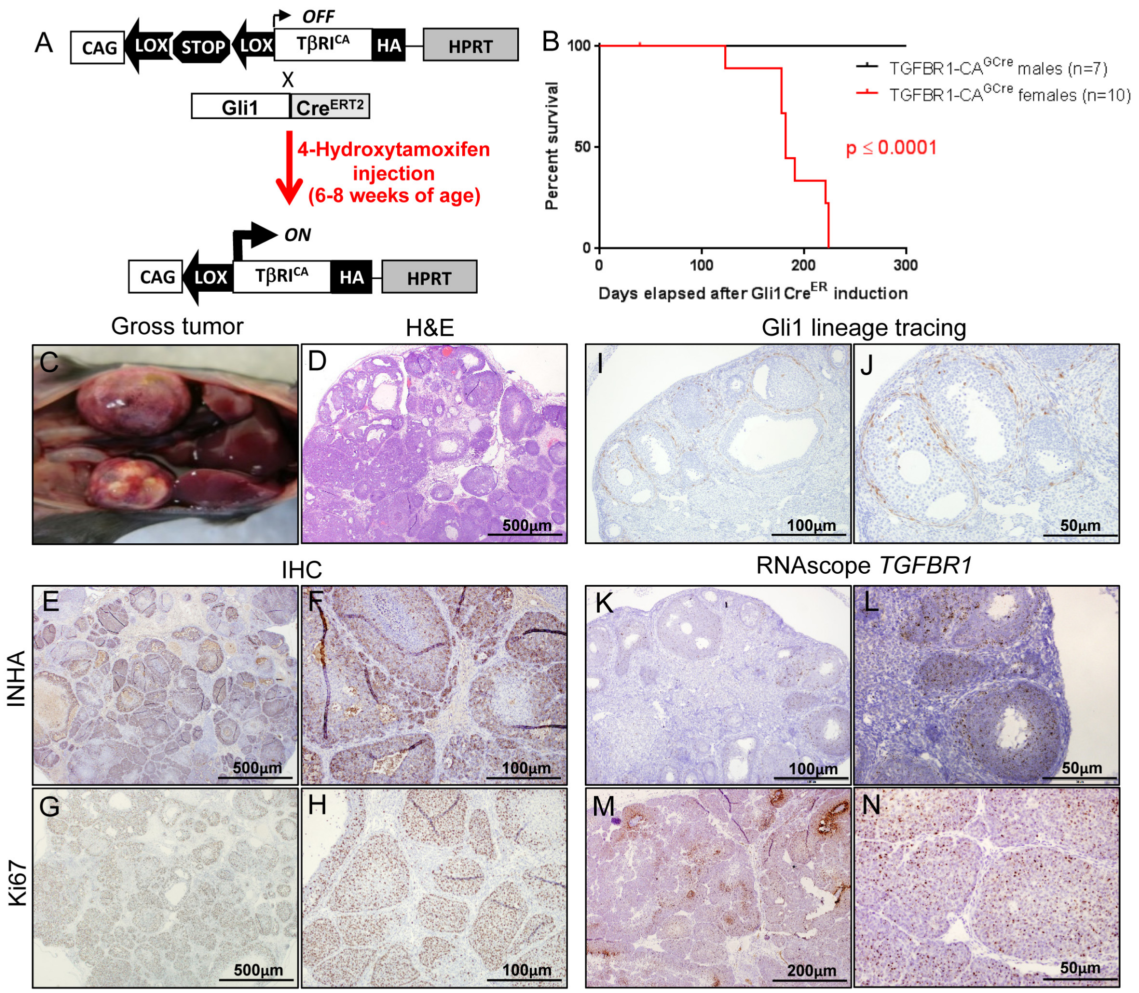

Figure S7. Constitutive activation of TGFBR1 using *Gli1*-Cre^{ERT2} in *Gli1*-positive population in adult mice leads to ovarian tumor development. (**A**) Schematic representation of the generation of *Gli1*-Cre^{ERT2} x LSL-TGFBR1^{CA} model (TGFBR1-CA^{Gcre}). (**B**) Kaplan-Meier curve comparing TGFBR1-CA^{Gcre} male (n = 7) and female (n = 10) survival after *Gli1*-Cre^{ERT2} induction. Note that only females developed a phenotype and succumbed to death. Mantel-Cox statistical test was performed. (**C** and **D**) Representative macroscopic and H&E staining images of ovarian tumors developed in TGFBR1-CA^{Gcre} female mice at 6 months of age. (**E-H**) Immunohistochemical staining of INHA (E and F) and Ki67 (G and H) using TGFBR1-CA^{Gcre} tumor tissues at 6 months. (**I** and **J**) Immunohistochemical analysis of RFP expression 7 days after *Gli1*-Cre^{ERT2} induction in mice harboring the LSL-RFP reporter gene. (**K-N**) Representative images of in situ localization of TGFBR1^{CA} mRNA using RNAscope 4 days (K and L) or 8 months (M and N) after TGFBR1^{CA} induction. Panels (F, H, J, L, and N) represent higher power images for panels (E, G, I, K, and M). Scale bars are shown in (D-N).

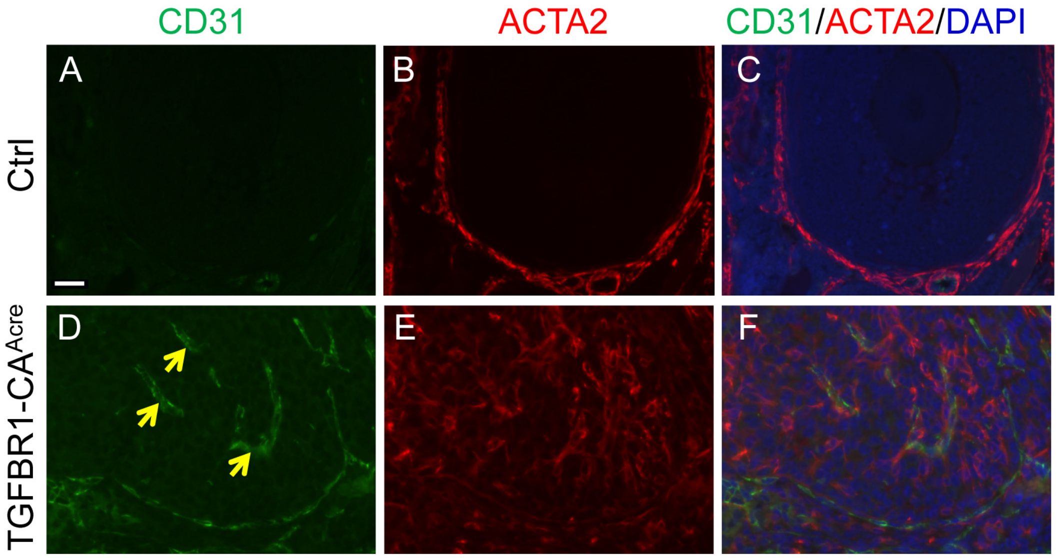

Figure S8. Evidence of angiogenesis in ovarian tumors from TGFBR1-CA^{Acre} mice. (**A-C**) Double immunofluorescence of CD31 and ACTA2 in 2-month-old control ovaries. Representative images show a normal follicle that expresses ACTA2 (red; B and C) in the theca layer. No CD31 expression (green; A and C) within the granulosa cell layers was detected. (**D-F**) Double immunofluorescence of CD31 and ACTA2 in 2-month-old TGFBR1-CA^{Acre} ovaries. Note the presence of blood vessels within the follicle-like structures of the TGFBR1-CA^{Acre} ovaries (Arrows). Scale bar is representatively shown in (A) and equals 20 μm (A-F). Immunofluorescence was performed using ovarian samples from 6 mice (n = 3 per group).

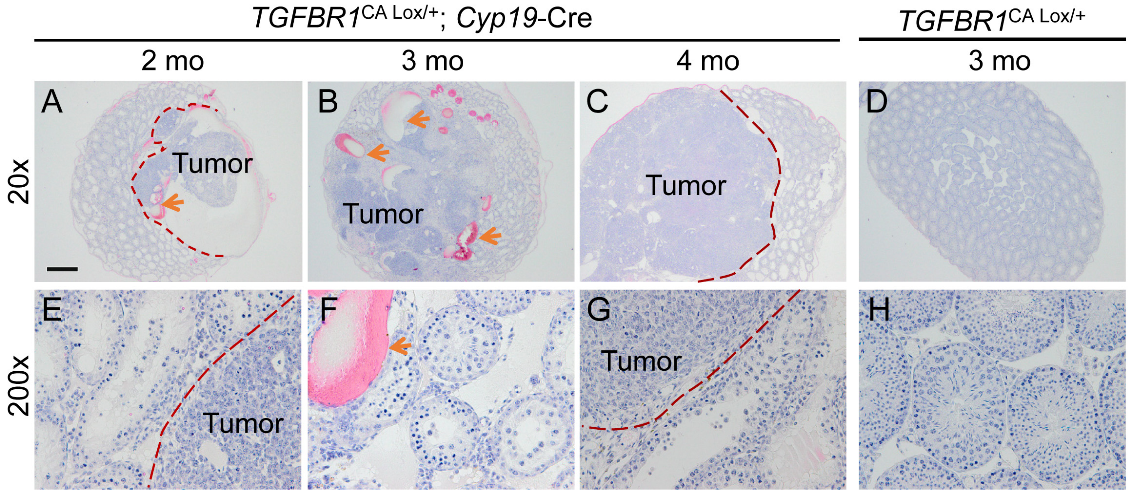

Figure S9. *TGFBR1*$^{\mathrm{CA\ Lox/+}}$; *Cyp19*-Cre males develop testicular tumor. (**A-H**) Histological analysis of tumor development in *TGFBR1*$^{\mathrm{CA\ Lox/+}}$; *Cyp19*-Cre males. Panels (E-H) represent higher power images for panels (A-D), respectively. Note testicular tumors at 2 (A and E), 3 (B and F), and 4 (C and G) months of age compared with representative controls (D and H). Arrows indicate hemorrhage (A, B, and F). Dashed lines demarcate the boundary between tumors and abnormal seminiferous tubules (A, C, E, and G). H&E staining was performed using mice at the age of 2-5 months (n = 5 for controls and n = 8 for *TGFBR1*$^{\mathrm{CA\ Lox/+}}$; *Cyp19*-Cre mice). Scale bar is representatively depicted in (A) and equals 50 µm (E-H) and 500 µm (A-D).

Supplementary References

1. Bell JR, Mellor KM, Wollermann AC, Ip WTK, Reichelt ME, Meachem SJ, Simpson ER and Delbridge LMD. Aromatase deficiency confers paradoxical postischemic cardioprotection. Endocrinology. 2011; 152(12):4937-4947.

2. Segers I, Adriaenssens T, Wathlet S and Smitz J. Gene expression differences induced by equimolar low doses of LH or hCG in combination with FSH in cultured mouse antral follicles. J Endocrinol. 2012; 215(2):269-280.

3. Gao Y, Wen H, Wang C and Li Q. SMAD7 antagonizes key TGFbeta superfamily signaling in mouse granulosa cells in vitro. Reproduction. 2013; 146(1):1-11.

4. Gao Y, Duran S, Lydon JP, DeMayo FJ, Burghardt RC, Bayless KJ, Bartholin L and Li Q. Constitutive activation of transforming growth factor Beta receptor 1 in the mouse uterus impairs uterine morphology and function. Biol Reprod. 2015; 92(2):34.

5. Gao Y, Bayless KJ and Li Q. TGFBR1 is required for mouse myometrial development. Mol Endocrinol. 2014; 28(3):380-394.

6. Reardon SN, King ML, MacLean JA, Mann JL, DeMayo FJ, Lydon JP and Hayashi K. Cdh1 is essential for endometrial differentiation, gland development, and adult function in the mouse uterus. Biol Reprod. 2012; 86(5):1-10.

7. Mariani J, Favaro R, Lancini C, Vaccari G, Ferri AL, Bertolini J, Tonoli D, Latorre E, Caccia R, Ronchi A, Ottolenghi S, Miyagi S, Okuda A, Zappavigna V and Nicolis SK. Emx2 is a dose-dependent negative regulator of Sox2 telencephalic enhancers. Nucleic Acids Res. 2012; 40(14):6461-6476.
